# Making the most of common impact metrics: promising approaches that need further study

**DOI:** 10.1186/1471-2458-13-S2-S8

**Published:** 2013-06-17

**Authors:** John Grove, Joseph W Brown, Philip W Setel

**Affiliations:** 1 Bill and Melinda Gates Foundation, 500 Fifth Avenue North, Seattle, WA 98102 USA

## 

There is considerable value in exploring whether and how a limited set of common health metrics, such as the ones presented in this supplement, can produce valid and reliable estimates of impact for global health and family planning. Through a shared vocabulary of transparent data elements drawn from routine service statistics, population-level measures, and measures of proven effect from rigorous trials, such metrics could provide a view of collective impact sufficient to reliably explore scenarios, set priorities, and inform action.

This volume represents an important step in the right direction. The remainder of our commentary argues the case for continued work toward valid, common impact metrics and some other ways in which common metrics can contribute to our work. Specifically: more cost-efficient monitoring; better collective decision making; transparency and alignment; and enhanced accountability.

Although we are enthusiastic about this line of scientific work and support the approach to planning, assessment, and performance management reflected in the papers here, there are some important caveats. While keeping the promise of modeled estimates in view, we believe more research into the validity and potential sources of bias and uncertainty in modeled impacts, and in the underlying source data, is required.

## More cost-efficient monitoring

Assuming that routine collection and analysis of service provision data are strong, and that data are verified and robust, there are potential time and resource savings that modeled impact efforts offer. One widely-used global tool that illustrates the potential of this approach is the Lives Saved Tool or 'LiST' [1]. By using a variety of data sources to estimate potentially avertable child mortality burdens under differing scenarios of intervention coverage (which LiST users design and manipulate), the tool aids impact-oriented planning and priority setting.

Various approaches to local level estimation could supplement, and in some cases replace, what has become a nearly exclusive reliance on large-scale surveys. Many of these surveys are not powered to produce reliable, current impact estimates at the sub-national level. Resources that might otherwise be put into more surveys could be redirected to build local measurement capacity and improve critical service delivery for populations in need. Savings might also be invested in initiatives that could improve the efficiency and quality of data collection, processing, and analysis. These efforts could include the development of human capacity, technological improvements, and the further exploration of methodological innovations.

## Better decision making

Collective measurement efforts are needed to utilize common metrics to improve decision making globally and locally. The data collection and analysis processes described in this volume encourage data sharing and accounting for the contribution of various actors in an open way. Common metrics should represent the basis for collaboration among donors, experts, and researchers, and users of data to identify gaps in service and determine how best to address them. Collaborative structures with clear remits to support policy decisions or guidance - such as Monitoring and Epidemiology Reference Groups ('MERGs') or the Essential Medicines Monitoring & Evaluation Working Group - convene technical experts, donors, and service agencies. These bodies can effectively promote and create a demand for continuous review and evaluation of methodological innovation globally; they can elevate the agenda on the importance of alternative approaches to data collection and on enhancing the reliability of metrics.

Common metrics alone do not ensure quality decisions on a global level, nor do they guarantee local services will be optimized. These metrics are necessary but not sufficient. The most they can offer is an empirical platform on which to explore a broader set of issues and trade-offs as decision makers seek to maximize value for the beneficiaries of health services. Intervention choices are also influenced by local context - ranging from political values, social enfranchisement, judgment, ethics, and technical and operational feasibility. It is hoped that routine data and impact estimates will be considered together with a frank review of contextual program, operational, cultural, and policy realities. For these efforts to be successful, a mix of strong local capacity and global support for local solutions is necessary.

## Transparency and alignment

Common metrics are essential for providing cohesion around goals and objectives for complex global initiatives. An example is the July 11, 2012 "London Summit on Family Planning" (FP2020), which effectively moved family planning back into the center of the global development and public health agenda [2]. The goal around which donors, multilateral organizations, national governments, and civil society organizations rallied was ambitious: to ensure that between 2012 and 2020, at least 120 million more women in 69 low-income countries will be using modern methods of contraception. For the measurement community, such a goal comes with significant challenges.

Ensuring accountability in reporting results is one key challenge posed by this alignment around the FP 2020 goal. Such accountability is a key aspect of common metrics, as consensus around good measurement implies recognition on the part of stakeholders that they are accountable for results. How can a measurement plan lead to effective ways to promote accountability? We have seen examples of plans that emphasize transparency of data and reporting, so that the posted results of one implementing organization or geographical unit are available for all others to see. For a project in which multiple implementing sources submit results to an electronic data platform using common metrics, this platform would allow full viewing access and privileges for all who log on. The approach presented by Marie Stopes International in this volume uses this type of platform. The transparency associated with this approach, and the accountability engendered by it, should stimulate greater attention to data quality and concern for accurately measuring how goals and objectives are being met.

## Enhanced accountability

Stakeholder collaboration in the implementation of common metrics and their use for decision making can also enhance accountability in global health programs. Some recent initiatives offer models that are instructive.

The African Leaders Malaria Alliance (ALMA) established an accountability and transparency framework to track progress, facilitate a rapid response to emerging issues and bottlenecks in service delivery, and allow for the use of its scorecard for better collective learning. The 2011 ALMA Scorecard for Accountability and Action consists of a semi-automated database that tracks progress across key indicators covering malaria policy, financing, intervention coverage, and impact. The scorecard also includes tracer maternal and child health metrics. Country progress against each indicator is tracked regularly and color-coded using the common green-yellow-red traffic light system.

The scorecard posts monthly progress updates using data from sources that stakeholders generally consider standard and legitimate, such as data from WHO, the World Bank, and the Roll Back Malaria secretariat. The scorecard is built around common metrics and populated with data that are sourced from global institutions known for their rigorous engagement and capacity at the local level. Another aspect of ALMA is that the quarterly country reports are designed to highlight progress and successes, identify constraints, and recommend steps to address these constraints. ALMA then supports countries in finding appropriate solutions. In short, the ALMA scorecard is not a punitive grading system. The reliable data used to support the ALMA scorecard and the agreement that the scorecard's purpose is for collective action against malaria appear to be positive steps toward increased accountability.

The Every Woman Every Child global initiative also developed a forward-looking structure around indicators, data reporting, and accountability. Their Commission on Information and Accountability for Women's and Children's Health is a model for how a large global program can build governance and program improvement around data quality and accountability [3].

## Conclusion

We applaud the effort to develop common metrics of avertable burden to inform decision making. It is an important effort for the health metrics community to explore. Arriving at a set of methods and measures that balance scientific rigor, pragmatism, and the urgent need for better data at the local level is no small undertaking, but the journey has just begun. Investments in methodological innovation and improved capacity to manage data "at the source" are now more important than ever before.

From a technical perspective, more validation studies are needed before modeled impacts can be considered as credible evidence of attributable or actual impact, and the wholesale use of modeled outputs alone in such an evaluative manner seems to us premature. For example, before we can be confident in the impact estimates generated, we must be confident they are good at overcoming the most vexing problem in global monitoring and evaluation: accurately assessing or estimating intervention coverage. While a good measure or approximation of intervention coverage is essential to estimating impact, the data needed to support coverage indicators are often difficult to collect through observational techniques, and operational definitions of key terms (e.g., "delivery with a skilled birth attendant") often vary by country or program.

Moving forward, donors and partners need to continue to integrate efforts and prioritize common metrics whenever possible. Pushing for innovation in method and efficiencies would improve our assessment tools and processes - as well as our decision making - even further. Data sharing, transparency, and peer review are critical in this new context.

## Disclaimer

The views expressed are those of the authors alone and do not necessarily reflect an official position of the Bill and Melinda Gates Foundation.

## List of abbreviations used

LiST: Lives Saved Tool; MERGs: Monitoring and Epidemiology Reference Groups; FP2020: 2012 London Summit on Family Planning; ALMA: African Leaders Malaria Alliance; WHO: World Health Organization

## Competing interests

The authors declare that they have no competing interests.

## Declarations

This article is part of the supplement of *BMC Public Health *Volume 13, Supplement 2, 2013: Use of health impact metrics for programmatic decision making in global health. Population Services International, a registered non-profit organization, provided the funding for the publication of this supplement. The full contents of the supplement are available online at http://www.biomedcentral.com/bmcpublichealth/supplements/13/S2.
